# *PagIPT5* Mediates Cambial Development in Poplar via Cytokinin–Auxin Crosstalk

**DOI:** 10.3390/genes17070756

**Published:** 2026-06-30

**Authors:** Yuhan Chen, Xiaoxue Hong, Jianyu Gu, Xin Tian, Xianghong Li, Xinyu Zhang, Yi An, Cheng Jiang, Ningning Chen, Hui Wang, Mengzhu Lu, Jin Zhang, Lichao Huang

**Affiliations:** National Key Laboratory for Development and Utilization of Forest Food Resources, Plant Cell Wall Research Centre, College of Forestry and Biotechnology, Zhejiang A&F University, Hangzhou 311300, China

**Keywords:** vascular cambium, tension wood, antagonism, cytokinin, auxin

## Abstract

Background/Objectives: Cytokinin and auxin are essential for vascular development in plants. This study aims to explore whether these two hormones exhibit crosstalk in the cambium, analogous to that observed in the apical meristem. Methods: Using the hybrid poplar (*Populus alba × P. glandulosa* clone ‘84K’), we integrated gravitropic induction with transcriptomic analysis and identified the cytokinin biosynthesis gene *PagIPT5* as differentially expressed in a tension wood induction system. *PagIPT5* overexpression lines were generated and assessed for growth-related phenotypes. The interaction between cytokinin and auxin was investigated via anatomical observation, cell proliferation assays, *in situ* PCR, and immunofluorescence detection of auxin and cytokinin. Results: Compared with the wild type, *PagIPT5* overexpression lines showed growth inhibition and an auxin-deficient phenotype. High *PagIPT5* expression in the vascular cambium elevated cytokinin levels while reducing auxin levels, leading to decreased cambial cell proliferation and suppressed xylem development. However, in the tension wood induction system, both auxin and cytokinin levels increased in the vascular cambium of tension wood relative to opposite wood. Treatment with a superoxide anion activator promotes the accumulation of both auxin and cytokinin in 84K plants. Conclusions: These results revealed an antagonistic interaction between the two hormones in the cambium zone. However, this antagonism is attenuated in tension wood, which may be induced by the accumulation of superoxide anion in tension wood.

## 1. Introduction

The vascular cambium is a secondary meristem formed through the dedifferentiation of mature parenchyma cells [1,2]. Its initiation and development play a critical role in plant secondary growth, as most tree structures are derived from cambial activity [3]. The secondary xylem (wood) originating from vascular cambium cell division and differentiation in tree stems is an important renewable biomass resource. Understanding the regulatory mechanisms of vascular cambium cell activity and using this knowledge for targeted improvement of plant traits is an effective way to enhance wood yield and quality.

Vascular cambium activity is regulated by multiple factors, including the class III homeodomain-leucine zipper (HD-ZIP III) transcription factor family member [4,5] and the CLE (CLAVATA3/EMBRYO SURROUNDING REGION-RELATED)-PXY (PHLOEM INTERCALATED WITH XYLEM)-WOX (WUSCHEL-RELATED HOMEOBOX) signaling pathway [6]. In addition, hormones play very important roles in regulating cambial cell proliferation and differentiation [7,8]. Studies have shown that disrupting auxin transport leads to decreased cambial cell proliferation activity, along with a significant reduction in the radial diameter and length of differentiated xylem fiber cells [9]. In *Arabidopsis*, gibberellin can regulate stem cell fate decisions during cambial proliferation, adjusting the ratio of xylem to phloem by influencing the differentiation direction of cambial stem cells [8]. Ethylene plays a dual role in the vascular cambium: regulating cambial cell number while promoting xylem vessel differentiation [10].

During plant vascular tissue development, cytokinin mainly accumulates in the vascular phloem but is also involved in regulating cambial activity [11]. Members of the isopentenyltransferase (IPT) family serve as key rate-limiting enzymes in cytokinin biosynthesis [12]. In *Arabidopsis*, there are two types of isopentenyltransferases: ATP/ADP isopentenyltransferases (AtIPT1, 3, 4–8) and tRNA isopentenyltransferases (AtIPT2, 9). Different types of isopentenyltransferases act on the synthesis of different cytokinin species [13]. ATP/ADP isopentenyltransferases participate in the methylerythritol phosphate (MEP) pathway to synthesize *trans*-zeatin (tZ), while tRNA isopentenyltransferases catalyze the synthesis of *cis*-zeatin (cZ) from dimethylallyl diphosphate (DMAPP). tZ and cZ can be interconverted and jointly participate in cytokinin biosynthesis [12]. Studies in *Arabidopsis* [14] and poplar [15] have shown that cytokinin regulates shoot apical meristem activity and lateral branch initiation through interaction with auxin. However, whether a similar interaction between the two hormones in the vascular cambium remains to be investigated.

Under gravitational or mechanical stress, the upper side of bent stems in woody plants produces a specialized xylem tissue called tension wood (TW). Its characteristics include an increased cambial cell proliferation rate, reduced vessel number, and the formation of a gelatinous layer on the inner side of xylem fiber cell walls. The vascular tissue morphology on the lower side of the bent stem is similar to that of normally growing plants and is called opposite wood (OW) [16]. Due to the differences in vascular cambium cell proliferation and differentiation patterns between tension wood and opposite wood, the tension wood induction system provides an ideal model for studying vascular tissue growth and development in woody plants. In this study, hybrid poplar (*Populus alba × P. glandulosa* clone ‘84K’) was subjected to gravitropic induction. Subsequent transcriptomic analysis revealed that *PagIPT5*, which encodes an IPT family member, is differentially expressed in the tension wood induction system. We further generated *PagIPT5* overexpression transgenic plants. Through growth index measurements, vascular tissue morphological observation, target gene expression pattern analysis, and auxin level detection in wild-type and overexpression plants, we aimed to explore the interaction between cytokinin and auxin in the vascular cambium of woody plants.

## 2. Materials and Methods

### 2.1. Plant Materials

Wild-type (WT) *P. alba* × *P. glandulosa* clone ‘84K’ was originally sourced from South Korea in 1984 and has since been widely grown across northern China. The material used in this study was maintained and propagated via tissue culture at 25 °C under a 16-h light/8-h dark photoperiod. The leaves of these tissue-cultured plants can be further used for genetic transformation. For physiological tests, 30-day-old tissue-cultured WT plants or transplants were transferred into soil and grown under the same temperature and light conditions.

### 2.2. Bioinformatics Analysis of the Poplar IPT Gene Family

Protein sequence information for *Arabidopsis* and *Populus trichocarpa* IPT gene family members was obtained from the PlantGenIE database (https://plantgenie.org/, accessed on 27 August 2024). A phylogenetic tree was generated with MEGA software using the maximum likelihood method. Protein sequence information was uploaded to MEME (https://meme-suite.org/meme/, accessed on 27 August 2024) to obtain motif information, which was imported into TBtools (v0.66673) for visualization.

### 2.3. Vector Construction

Total RNA was extracted from fresh wild-type plant leaves using an RNA extraction kit (RN35, BIOFIT, Shanghai, China) and reverse transcribed into cDNA using a reverse transcription kit (AG11711, Accurate Biology, Hunan, China). The target gene CDS was amplified by PCR with specific primers. The pMDC43 vector was linearized by KpnI/SacI double digestion for the construction of *35S:PagIPT5*, and by AscI/PacI double digestion for the construction of *35S:PagIPT5-GFP*. Vectors were constructed through homologous recombination (C115, Vazyme, Nanjin, China). The primers used are listed in Supplementary Table S1.

### 2.4. Plant Transformation

The successfully verified *35S:PagIPT5* vector was transformed into *Agrobacterium tumefaciens* strain GV3101 competent cells. Leaves from 3-week-old 84K poplar tissue-cultured plants were cut and immersed in an *Agrobacterium* suspension carrying the overexpression vector for 15 min. The infected leaves were placed on co-cultivation medium and cultured in the dark at 25 °C for 2 d, then sequentially transferred to differentiation medium to induce adventitious shoots and rooting medium to induce roots.

### 2.5. Subcellular Localization Analysis of the PagIPT5 Protein

The *35S:GFP-PagIPT5* construct was transformed into GV3101 competent cells for transient infiltration into mesophyll cells of 4-week-old *Nicotiana benthamiana* plants. GFP fluorescence signals were observed using a confocal laser scanning microscope (LSM880, Zeiss, Jena, Germany).

### 2.6. Phenotypic Observation of PagIPT5 Overexpression Plants

Thirty-day-old tissue-cultured wild-type and *PagIPT5* overexpression plants were sampled. The 9th internode stem segment was sectioned (50 μm) using a vibratome (VT1200, Leica, Wetzlar, Germany). For vascular tissue morphology, sections were stained using 0.1% toluidine blue O (TBO) and examined under an upright fluorescence microscope (DM6B, Leica). A plant image analyzer (S20200222, WSeen, Hangzhou, China) was used to scan plant roots and analyze root-related traits. The 5th, 7th, and 10th internodes of 45-day-old soil-grown wild-type and *PagIPT5* overexpression plants were harvested and sectioned (50 μm) for histochemical staining with 0.1% TBO, 1% (*w*/*v*) phloroglucinol/HCl, and 0.01% Calcofluor white (CFW, 18909, Sigma-Aldrich, St. Louis, MO, USA) to observe vascular tissue morphology and qualitatively analyze xylem cell wall components.

### 2.7. In Situ PCR Analysis of PagIPT5 Gene Expression Pattern in Vascular Tissues

The 9th internode stem segments from 30 d *PagIPT5* overexpression lines and wild-type tissue-cultured plantlets were fixed (63% ethanol, 5% acetic acid, 2% formaldehyde) and sectioned (50 μm). Genomic DNA was removed by DNase I (2270A, Takara, Kusatsu, Japan) treatment, and cDNA was synthesized by reverse transcription using SMART MMLV Reverse Transcriptase (639523, Takara). *In situ* PCR was performed using PrimeSTAR^®^ Max (R047A, Takara) and DIG-11-dUTP (DIUTPS-RO, Roche, Mannheim, Germany). After washing with 1× PBS, sections were blocked with 1% BSA for 30 min. Subsequently, sections were incubated with Anti-Digoxigenin-AP antibody (11093274910, Roche) (1:500) for 1 h, and washed with washing buffer (0.1 M Tris-Cl, 0.15 M NaCl, pH 9.5). Color development was performed using BM purple (11442074001, Roche) for 10 min, and observations were made with an upright fluorescence microscope (DM6B, Leica) [17].

### 2.8. Immunofluorescence Assay

Stem segments from specific materials were fixed in 4% paraformaldehyde and cut into 100-μm sections. Sections were incubated in 1× PBST for 30 min. After blocking with 5% BSA for 1 h, sections were incubated with primary antibody overnight at 4 °C. After washing with 1× PBS, sections were incubated with DyLight488-conjugated goat anti-rabbit secondary antibody (AS09633, Agrisera, Vännäs, Sweden) (1:200) for 1 h, washed with 1× PBS, and observed under an upright fluorescence microscope (DM6B/DM6B Thunder, Leica). Rabbit anti-IAA (AS09421, Agrisera) was used at a 1:100 dilution as the primary antibody for auxin level detection; Rabbit anti-*trans*-zeatin riboside (AS09429, Agrisera) and Rabbit anti-N6-isopentenyladenosine (AS09435, Agrisera) were used at a 1:200 dilution as primary antibodies for cytokinin level detection.

### 2.9. Detection of Vascular Cambium Cell Proliferation Rate

The 7th internode of 45 d soil-grown wild-type and *PagIPT5* overexpression plants was treated with a mixture of 20 μM F-*ara*-EdU and lanolin paste, followed by 4 days of continued growth. Three days after removing the lanolin, the treated internodes were collected, fixed, and sectioned (50 μm). The BeyoClick™ EdU-488 Cell Proliferation Assay Kit (C0071S, Beyotime, Shanghai, China) was used to analyze the retained F-*ara*-EdU fluorescence signal in the samples. DAPI staining was performed, and an upright fluorescence microscope (DM6B, Leica) was used to observe and quantify the ratio of the number of F-*ara*-EdU-labeled DNA to the number of DAPI-labeled DNA [18].

### 2.10. Expression Pattern Analysis of IPT5 Family Members in Tension Wood

The materials used for transcriptome sequencing were obtained from nine plants subjected to gravistimulation for 48 hours. Samples from every three plants were pooled into one group, resulting in a total of three biological replicates. The middle internode of the bent stem from plants subjected to gravistimulation was dissected into TW and OW fractions. After removing the bark, the cambium and developing xylem tissues were gently scraped and collected for RNA extraction, library construction, and sequencing, following the protocol described by Cheng et al. [19]. Differentially expressed genes (DEGs) were identified by DESeq2 software based on a fold-change threshold > 1.5 and an FDR-adjusted *p* < 0.05. A heatmap was constructed using the HeatMap function of TBtools, based on the FPKM (fragments per kilobase per million) values calculated for the IPT gene family.

## 3. Results

### 3.1. PagIPT5 Is Upregulated in Tension Wood

**Figure 1:**
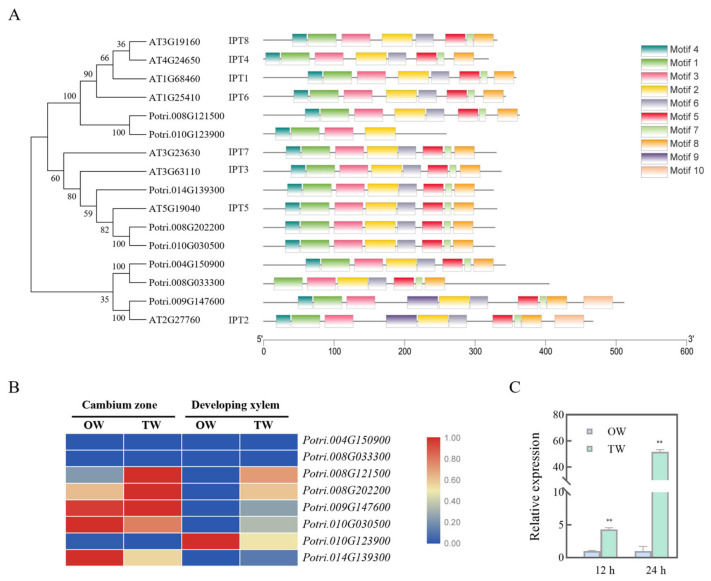
Bioinformatics analysis of the IPT gene family: (**A**) Evolutionary relationship of IPT gene family members from poplar and *Arabidopsis*. (**B**) Heatmap showing the expression levels of IPT gene family members in the tension wood induction system. (**C**) Expression analysis of *PagIPT5* in the tension wood system (*n* = 3). Data are represented as mean ± SD. ** *p* ≤ 0.01; Student’s *t*-test.

To screen for members of the poplar IPT gene family potentially involved in vascular cambium activity in forest trees, we constructed a phylogenetic tree showing the relationship between *Arabidopsis* and poplar IPT family members using the maximum likelihood method (Figure 1A). We then utilized transcriptomic data from the poplar TW induction system (a classic model for studying vascular cambium cell activity) to screen for IPT gene family members that are differentially expressed between OW and TW. Transcriptome data analysis showed that the homologous gene *Potri.008G202200* in 84K poplar was upregulated in TW (Figure 1B) and exhibited relatively high expression levels in vascular phloem (Figure S1), and it was named *PagIPT5* based on its position in the phylogenetic tree. Subcellular localization analysis showed that the PagIPT5-GFP fusion protein exhibited punctate distribution in the cytoplasm of tobacco mesophyll cells and was also localized in the nucleus (Figure S2). Quantitative real-time PCR (qRT-PCR) was performed to further validate its transcriptional levels in the tension wood system (Figure 1C). At 12 hours after gravity induction, the transcriptional level of this gene in TW was already significantly higher than that in OW. This difference was further amplified at 24 hours, with the transcriptional level in TW reaching 51.7 times that in OW. These results suggest that this gene may be involved in regulating vascular cambium activity.

### 3.2. PagIPT5 Overexpression Inhibits Auxin Levels in the Vascular Cambium

**Figure 2:**
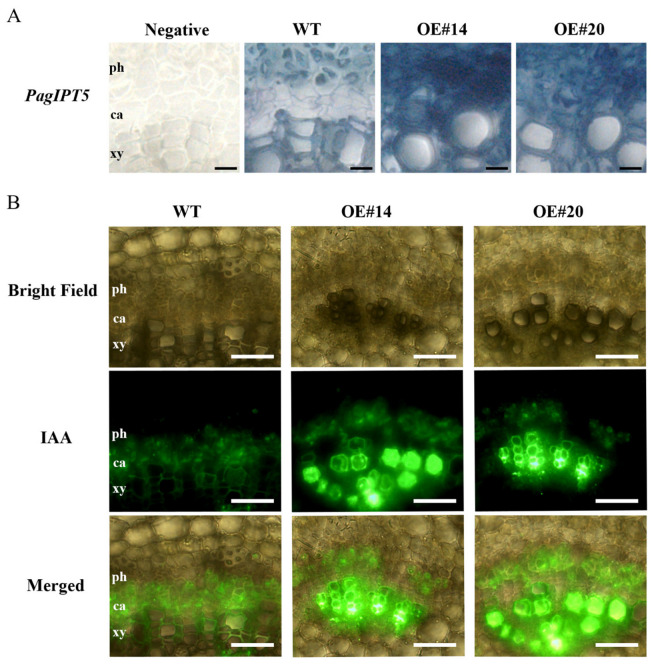
Anatomical observation of vascular tissues and physiological index analysis in *PagIPT5* overexpression plants: (**A**) *In situ* PCR assay detecting *PagIPT5* expression levels in vascular tissues of 30-day-old tissue-cultured transgenic and wild-type plants. Scale bar = 10 μm. (**B**) IAA immunofluorescence assay analyzing auxin levels in vascular tissues of 30-day-old tissue-cultured *PagIPT5* overexpression and wild-type plants. ph, phloem; ca, cambium; xy, xylem. Scale bar = 50 μm.

To investigate the role of *PagIPT5* on poplar vascular development, we generated *35S:PagIPT5* transgenic lines and selected overexpression lines (OE#14 and OE#20) where the target gene transcriptional levels were 58- and 45-fold higher than in the wild type, respectively, for subsequent functional analysis (Figure S3). Root growth parameters of 30-day-old tissue-cultured plants indicated that the total root length of transgenic plants was significantly reduced compared to the wild type, while the number of adventitious roots significantly increased (Figure S3), exhibiting phenotypic characteristics resembling auxin deficiency. Analysis of *PagIPT5* expression and auxin content in the vascular cambium region of transgenic plants using *in situ* PCR combined with IAA tissue immunofluorescence assays revealed that in tissue-cultured wild-type plants, the *PagIPT5* expression signal in the vascular cambium region was noticeably weaker than in the phloem and xylem, whereas in overexpression plants, the *PagIPT5* expression signal was broadly distributed in the phloem, cambium, and xylem, with significantly higher signal intensity in the cambium region than that in the wild type (Figure 2A). Correspondingly, IAA tissue immunofluorescence results showed that the IAA signal intensity in the cambium region of overexpression plants was significantly lower than that of the wild type (Figure 2B). These results indicate that overexpression of *PagIPT5* in the cambium region inhibits auxin levels in the cambium.

### 3.3. Vascular Cambium Activity Is Inhibited in PagIPT5 Overexpression Plants

**Figure 3:**
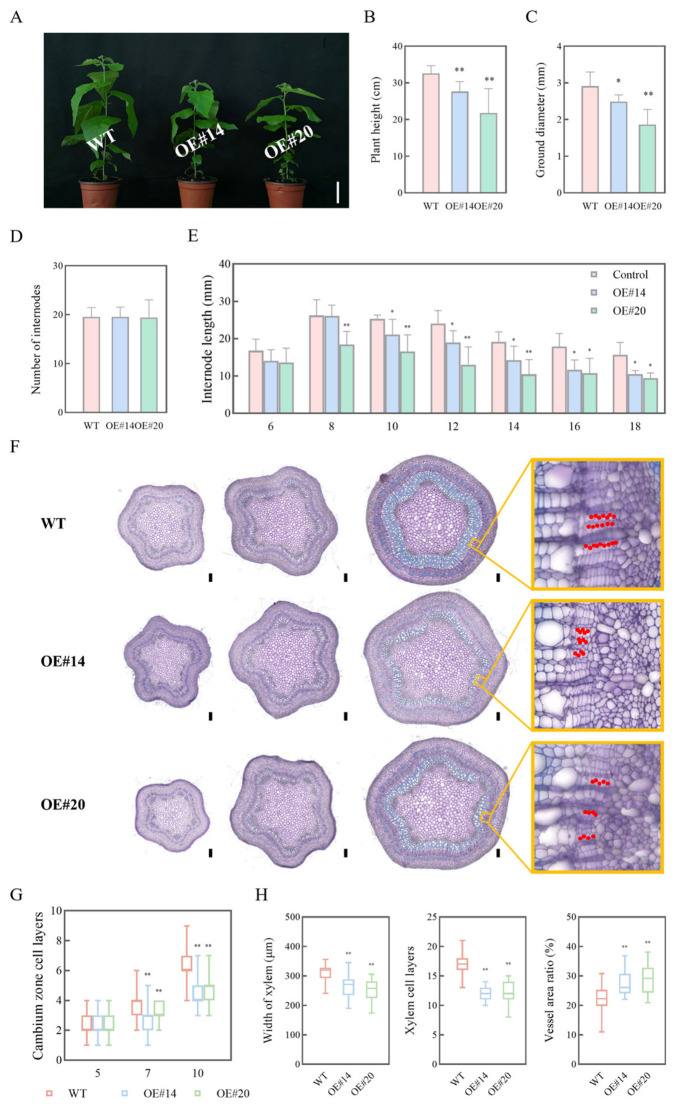
Phenotypic observation and vascular tissue anatomy of *PagIPT5* overexpression plants: (**A**) Phenotypes of *PagIPT5* overexpression and wild-type plants grown in soil for 45 days. Scale bar = 5 cm. (**B**–**E**) Quantification of plant height (*n* ≥ 6), ground diameter (*n* ≥ 5), internode number (*n* = 7), and lengths of the 6th, 8th, 10th, 12th, 14th, 16th, and 18th internodes (*n* ≥ 6) in *PagIPT5* overexpression and wild-type plants grown in soil for 45 days. (**F**) TBO histochemical staining of vascular tissues in the 5th, 7th, and 10th internodes of *PagIPT5* overexpression and wild-type plants grown in soil for 45 days. Scale bar = 250 μm. (**G**,**H**) Quantification of cambial cell layer number (*n* ≥ 90), xylem width, xylem (*n* ≥ 20) cell layer number (*n* ≥ 20), and vessel area percentage (*n* ≥ 20) in *PagIPT5* overexpression and wild-type plants grown in soil for 45 days. Data are represented as mean ± SD. * *p* ≤ 0.05; ** *p* ≤ 0.01; Student’s *t*-test.

**Figure 4:**
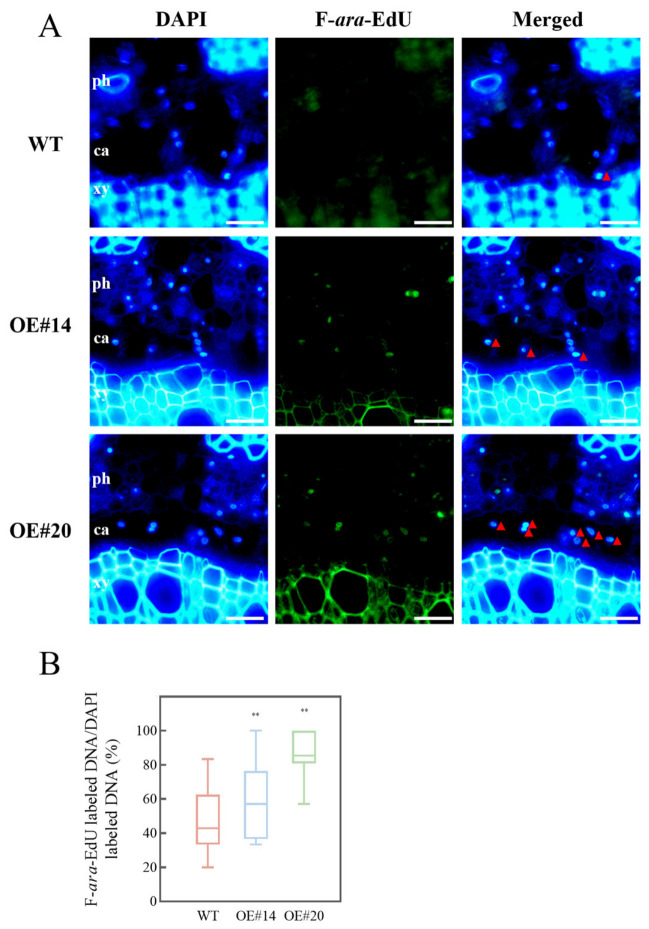
Detection of cambial cell proliferation rate in *PagIPT5* overexpression plants: (**A**) F-*ara*-EdU labeling assay analyzing the proliferation rate of cambial cells. Scale bar = 30 μm. (**B**) Quantification of the ratio of F-*ara*-EdU-labeled DNA to DAPI-labeled DNA (*n* ≥ 12). Data are represented as mean ± SD. ** *p* ≤ 0.01; Student’s *t*-test.

Growth index measurements and vascular tissue morphological analysis were conducted on 45-day-old soil-grown plants. The results showed that *PagIPT5* overexpression transgenic plants exhibited significantly reduced plant height and ground diameter compared to the wild type (Figure 3A–C). Further analysis of internode number and internode length revealed that the reduction in plant height in transgenic lines was attributable to a decrease in internode length (Figure 3D,E). Anatomical observation of stem vascular tissues showed that the number of vascular cambium cell layers, xylem width, and number of xylem cell layers were all significantly reduced in *PagIPT5* overexpression transgenic stems, while the vessel area ratio was significantly increased (Figure 3G,H). Qualitative analysis of xylem cell wall components showed weaker phloroglucinol staining and stronger CFW staining in the vascular xylem of overexpression lines, indicating decreased lignin content and increased cellulose content (Figure S4). These results suggest that cambial cell proliferation and differentiation processes may be inhibited in *PagIPT5* overexpression transgenic plants. Analysis of cambial cell proliferation rate using F-*ara*-EdU labeling showed that the retention ratio of F-*ara*-EdU in cambial cells of transgenic plants was significantly higher than in the wild type (Figure 4), indicating that high *PagIPT5* expression inhibits cambial cell proliferation.

### 3.4. PagIPT5-Mediated Cytokinin Accumulation Inhibits Auxin Levels in the Cambium

**Figure 5:**
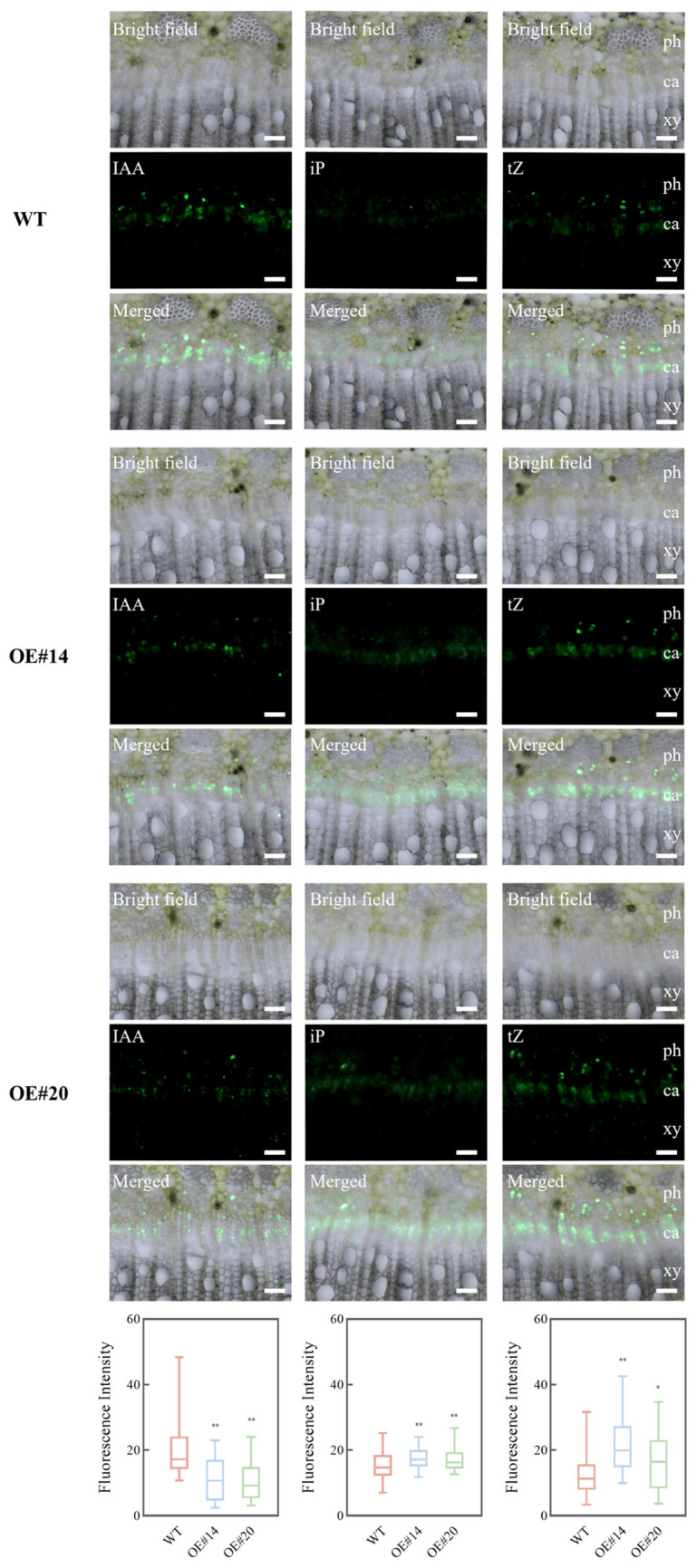
Hormone level analysis in the vascular cambium of *PagIPT5* overexpression plants. Immunofluorescence assay analyzing auxin and cytokinin levels in the vascular cambium of the 9th internode of *PagIPT5* overexpression and wild-type plants grown in soil for 60 days (*n* ≥ 27). ph, phloem; ca, cambium; xy, xylem. Scale bar = 50 μm. Data are represented as mean ± SD. * *p* ≤ 0.05; ** *p* ≤ 0.01; Student’s *t*-test.

Given that *PagIPT5* is a key gene involved in the cytokinin biosynthesis pathway, we performed tissue immunofluorescence assays to determine whether the reduced auxin levels in the vascular cambium of *PagIPT5* overexpression plants result from cytokinin accumulation. Specifically, we used tissue immunofluorescence assays to analyze the levels of cytokinin (iP and tZ) and auxin (IAA) in the vascular cambium of 60-day-old soil-grown transgenic lines. Tissue-cultured seedlings show retarded vascular development in overexpressing plants (no continuous cambial ring) (Figure 2B). Soil-grown seedling stem segments better reflect cambial hormone levels across lines than tissue-cultured ones. The results showed that cytokinin levels in the vascular cambium of *PagIPT5* overexpression plants were significantly higher than those in the wild type, but auxin levels were significantly lower (Figure 5). These findings indicate that *PagIPT5* overexpression-mediated cytokinin accumulation in the cambium zone leads to a decrease in auxin levels, revealing an antagonistic relationship between cytokinin and auxin in the vascular cambium region.

### 3.5. Antagonism Between Cytokinin and Auxin Is Weakened in the Vascular Cambium of the Tension Wood Induction System

**Figure 6:**
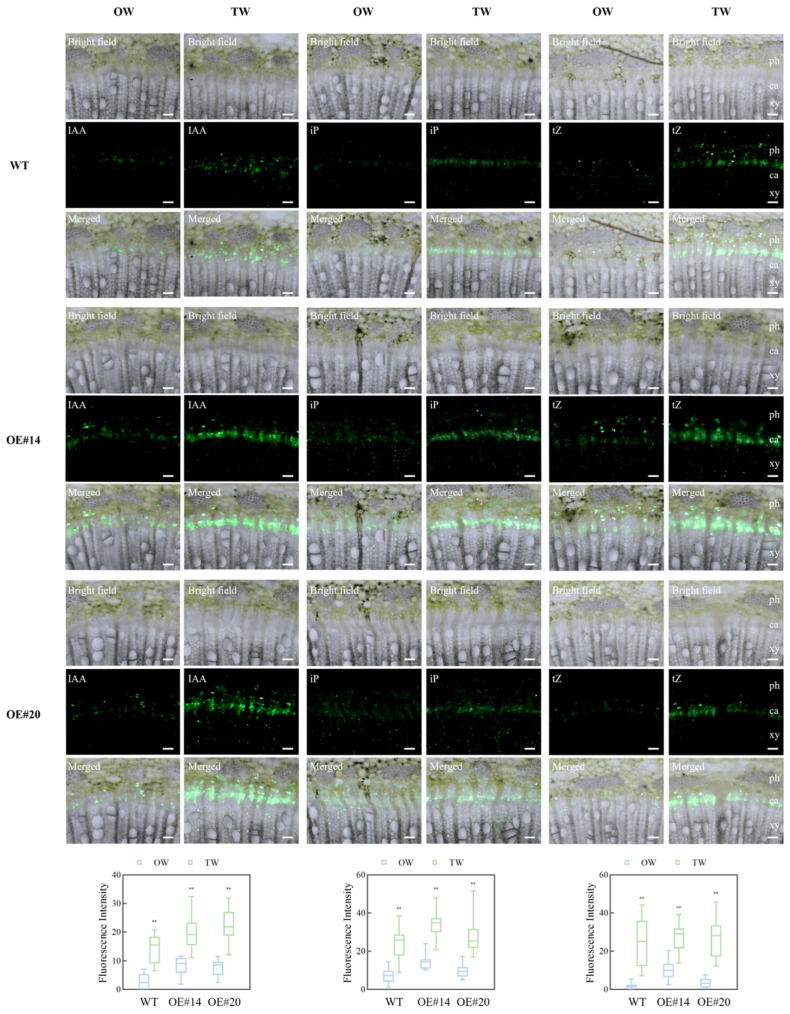
Analysis of auxin and cytokinin levels in the vascular cambium of *PagIPT5* overexpression plants after gravitropic induction treatment. Immunofluorescence assay analyzing auxin and cytokinin levels in the vascular cambium of TW and OW of *PagIPT5* overexpression and wild-type plants (*n* ≥ 12). ph, phloem; ca, cambium; xy, xylem. Scale bar = 50 μm. Data are represented as mean ± SD. ** *p* ≤ 0.01; Student’s *t*-test.

Previous studies have shown that under gravitropic induction conditions, superoxide anion content increases in the TW region of poplar, driving a significant increase in vascular cambium IAA levels compared to the OW region [18]. Our results also showed that the transcriptional level of *PagIPT5* in the TW region was significantly higher than in the OW region. To explore the relationship between cytokinin and auxin levels in the vascular cambium of the tension wood induction system, we further analyzed the contents of these two hormones in the TW and OW regions using tissue immunofluorescence assays (Figure 6). The results showed that in both wild-type and *PagIPT5* overexpression lines under 4 days of gravity induction, IAA levels in the TW region were significantly higher than in the OW region, consistent with previous findings [18]. However, the levels of iP and tZ, two cytokinins, were also significantly higher in the TW region compared to the OW region across different lines, indicating that the antagonism between cytokinin and auxin in the cambial zone of the tension wood induction system is disrupted.

**Figure 7:**
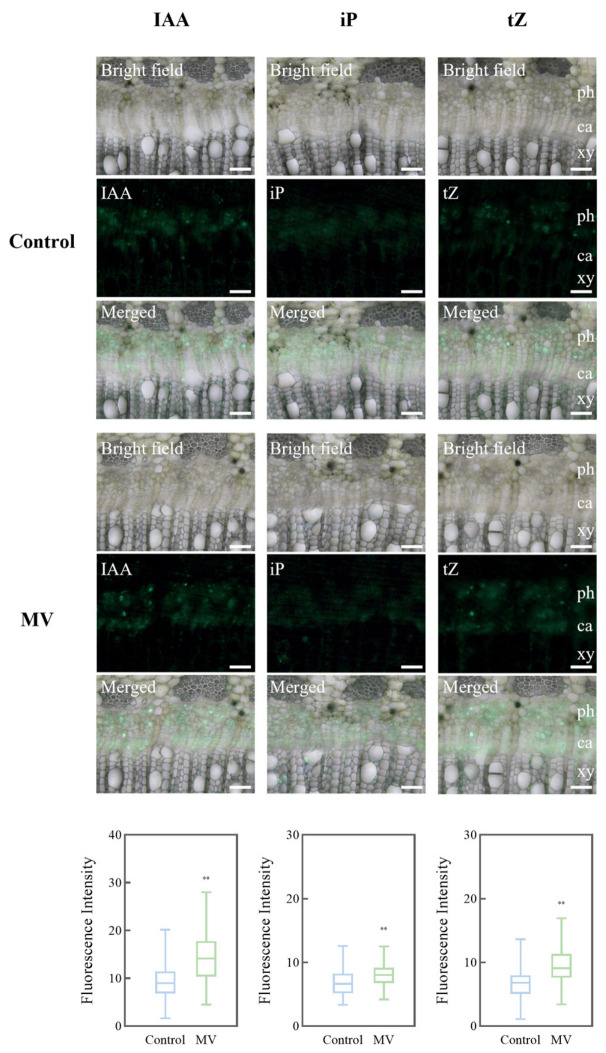
Analysis of auxin and cytokinin levels in the vascular cambium of poplar after MV treatment. Immunofluorescence assay analyzing the auxin and cytokinin levels in the vascular cambium of poplar after MV treatment (20 sections from four biological replicates were used, and fluorescence intensity was quantified at five randomly selected sites within the cambial zone per section using ImageJ software). ph, phloem; ca, cambium; xy, xylem. Scale bar = 50 μm. Data are represented as mean ± SD. ** *p* ≤ 0.01; Student’s *t*-test.

To further investigate the cause of this weakened antagonism in the tension wood system, we treated the 7th internode of 84K poplar with the superoxide anion activator methyl viologen (MV). After 4 days, we analyzed the levels of auxin and cytokinin in the vascular cambium region of the treated internodes. The results showed that IAA, iP, and tZ levels in the vascular cambium of the MV-treated group were all increased compared to the control group (Figure 7), indicating that MV-mediated elevation of superoxide anion levels can simultaneously promote the accumulation of both auxin and cytokinin in the poplar vascular cambium region. This suggests that superoxide anion accumulation in the TW cambium may be the reason for the weakened antagonism between cytokinin and auxin.

## 4. Discussion

Cytokinin plays a critical role in regulating the development of various plant meristems. In the shoot apical meristem, exogenous cytokinin treatment can induce meristem area expansion, and this effect is more pronounced in mutants of the secreted peptide-encoding gene *CLAVATA3* (*CLV3*). Further studies suggest that cytokinin may regulate the proliferative activity of stem cells in the shoot apex through antagonizing CLV3 signaling and inducing the expression of its downstream transcription factor *WUSCHEL* (*WUS*) [20]. In the root apical meristem, reducing root cytokinin levels delays cell differentiation, extends the proximal meristem zone, and thereby promotes root growth [21].

In the vascular cambium, cytokinin also serves as an essential signal for maintaining cell division activity. Concurrent knockout of four cytokinin biosynthesis genes, *IPT1*, *IPT3*, *IPT5*, and *IPT7*, leads to the loss of vascular cambium activity in *Arabidopsis*, with no secondary xylem formation in the hypocotyl [22]. The distribution of cytokinin in the vascular tissues of woody plants exhibits significant spatial specificity: its concentration peaks in the secondary phloem and decreases toward the vascular cambium and xylem. Disruption of this concentration gradient, either by phloem-specific expression of the poplar cytokinin degradation gene *CKX2* or by downregulation of the cytokinin receptor-encoding gene *HISTIDINE KINASE* (*HK*), results in inhibited vascular cambium cell proliferation [23]. Cambium-specific expression of the cytokinin degradation pathway oxidase gene *AtCKX2* in poplar leads to a reduced number of vascular cambium cell layers and a consequent significant decrease in trunk diameter [11]. In this study, overexpression of the *PagIPT5* gene led to a reduction in the number of vascular cambium cell layers (Figure 3F,G) and a decreased rate of cambial cell proliferation (Figure 4). Collectively, these findings indicate that cytokinin is required for the proliferation and differentiation of vascular cambium cells, but this function is limited to a specific concentration gradient range. Maintaining excessively high cytokinin concentrations in the cambium region instead inhibits cambial cell proliferation and differentiation.

Cytokinin and auxin engage in complex and dynamic interactions. Early studies on plant tissue culture revealed both antagonistic and synergistic interactions between these hormones. Exogenous auxin can induce cytokinin activity, and exogenous cytokinin also induces the expression of genes related to auxin biosynthesis and transport [24]. However, during the establishment of the shoot apical meristem in tissue culture, the two hormones act antagonistically: auxin restricts the expression range of the cytokinin biosynthesis pathway *AtIPT5* gene to the periphery of the auxin activity domain, thereby forming a corresponding cytokinin synthesis level gradient [25].

During the normal plant growth and development process, in the early stages of root apical meristem formation, auxin signaling directly activates the transcription of the cytokinin signaling feedback inhibitor-encoding genes *ARR7* and *ARR15*, thereby reducing cytokinin responsiveness in basal cells [26]. In the established root apical meristem, auxin signaling is concentrated in the quiescent center (QC), and cytokinin regulates auxin distribution levels in the QC by downregulating the expression of the auxin influx carrier protein-encoding genes *AUX1* and *LAX2*, thus regulating the proliferation and differentiation status of QC cells [27]. In the shoot apical meristem, cytokinin signaling is concentrated in the organizing center (OC), and auxin suppresses the cytokinin response pathway by activating the expression of *ABERRANT PHYLLOTAXY1* (*ABPH1*), which encodes a negative regulator of cytokinin signaling, thereby modulating the size of the shoot apical meristem [28]. Furthermore, during *Arabidopsis* floral meristem development, auxin activates the expression of the auxin response factor-encoding gene *ARF3*, which in turn represses the expression of IPT gene family members of the cytokinin biosynthesis pathway and the cytokinin receptor-encoding gene *ARABIDOPSIS HISTIDINE KINASE4* (*AHK4*), thus suppressing cytokinin activity [29]. In this study, we primarily explored the interaction between cytokinin and auxin in the woody plant vascular cambium meristem. The distribution pattern, in which auxin signaling is more concentrated in the vascular cambium and cytokinin signaling is more concentrated in the vascular phloem [23], resembles the auxin-cytokinin distribution pattern observed in roots [27]. In the *PagIPT5* overexpression lines, IAA levels in the vascular cambium were significantly decreased compared to the wild type, whereas tZ and iP levels were significantly increased (Figure 5), indicating antagonism between auxin and cytokinin in the vascular cambium region. The cytokinin accumulation mediated by *PagIPT5* in overexpression plants led to a decrease in auxin levels in the cambium zone, resulting in inhibited cambial cell proliferation (Figure 4). Constitutive overexpression of IPT in *Zanthoxylum piperitum* resulted in a significant decrease in auxin content in leaves [30]. In poplar, the auxin response factor PagARF3.1 represses cytokinin biosynthesis in roots by suppressing the expression of *PagIPT5a* and *PagIPT5b* [31]. This suggests that IPT genes may serve as a critical node in the auxin-cytokinin interaction network.

Previous studies have shown that gravitropic induction treatment leads to superoxide anion accumulation in the cambium zone of tension wood, which mediates an increase in auxin levels and consequently promotes cambial cell proliferation [18]. However, under gravitropic induction conditions, the transcriptional level of *PagIPT5* in the cambium zone of TW was significantly higher than that in OW (Figure 1C), suggesting that the tension wood cambium zone may still accumulate cytokinin under high IAA-level conditions. Gravitropic induction treatment was performed on transgenic plants, followed by tissue immunofluorescence analysis of IAA, tZ, and iP signals in vascular tissues. The results showed that IAA, tZ, and iP signals in the TW region of both wild-type and *PagIPT5* overexpression lines were higher than those in the OW region (Figure 6), indicating that the antagonism between auxin and cytokinin is attenuated in the vascular cambium of TW. Treatment with the superoxide anion activator MV, combined with tissue immunofluorescence analysis, revealed that MV treatment simultaneously promoted the accumulation of auxin and cytokinin in the vascular cambium zone (Figure 7). This suggests that superoxide anion may be a factor disrupting the antagonism between auxin and cytokinin in TW. Regarding this phenomenon in TW, we propose two hypotheses. Previous studies have found that outside the apical meristem, the antagonism between cytokinin and auxin is dose-dependent; when both hormones are present at high concentrations, the typical antagonism described above is no longer observed [32]. This raises the possibility that the attenuation of antagonism could also be caused by the high auxin accumulation mediated by superoxide anions. Alternatively, TW may balance its elevated auxin levels by mobilizing an increase in cytokinin levels.

## 5. Conclusions

In this study, transgenic lines overexpressing the cytokinin biosynthesis-related gene *PagIPT5* were generated in poplar. Through measurements of growth parameters, anatomical observation of vascular tissues, *in situ* PCR, and tissue immunofluorescence assays, we examined the interaction between cytokinin and auxin in the vascular cambium. The results showed that high-level expression of *PagIPT5* in the vascular cambium led to increased cytokinin levels, which in turn resulted in decreased auxin levels, a reduced cambial cell proliferation rate, and suppressed xylem development, revealing an antagonistic relationship between the two hormones in the vascular cambium. However, superoxide anion-mediated auxin accumulation in tension wood was able to disrupt this antagonism. These findings further our understanding of the molecular regulatory mechanisms governing the vascular cambium.

## Data Availability

The original contributions presented in this study are included in the article. For more information, please contact the corresponding author.

## Supplementary Materials

The following supporting information can be downloaded at: https://www.mdpi.com/article/10.3390/genes17070756/s1, Table S1: List of primers used in this paper; Figure S1: Expression of IPT gene family members in different tissues; Figure S2: Subcellular localization of the protein encoded by *PagIPT5*; Figure S3: Root growth parameters in *PagIPT5* overexpression plants; Figure S4: Qualitative detection of lignin and cellulose in *PagIPT5* overexpression plants. Reference [33] is cited in the Supplementary Materials.
