# Supplementary material for: PagIPT5 Mediates Cambial Development in Poplar via Cytokinin–Auxin Crosstalk
Source: Genes (Basel). 2026 Jun 30;17(7):756. doi: 10.3390/genes17070756 (PMC13408965; doi:10.3390/genes17070756)
Supplement: Supplementary file 1 [file genes-17-00756-s001.zip › genes-4372957-supplementary.pdf]

Supplementary Table S1. List of primers used in this paper

Primer name	Gene ID	Forward primer 5'→3'	Reverse primer 5'→3'	Notes
PagIPT5-OE	*Potri.008G202200*	ctagaggatccccgggtaccATGACCATGAGGCTTTCTTTG	gatcggggaaattcgagctcTTATCGACTAGCGACGGC	Cloning
PagIPT5-GFP	*Potri.008G202200*	atgaactatacaaaggcgcgccaATGACCATGAGGCTTTCTTTG	ccgctctagaactagttaatTTATCGACTAGCGACGGC	Cloning
PagIPT5-RT	*Potri.008G202200*	CAGCCTCGTGTGAATTTCCA	GCTTCCGTCACCTTGTTTGT	Realtime
PagACTIN-RT	*Potri.001G309500*	AAACTGTAATGGTCCTCCCTCCG	GCATCATCACAATCACTCTCCGA	Realtime

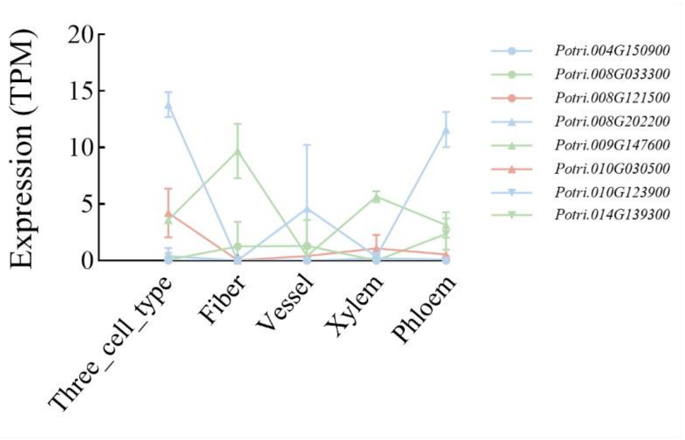

Supplementary Figure S1. Expression of IPT gene family members in different tissues
Visualization of IPT gene family expression in three type cell (fiber, vessel and ray cells), xylem fiber, xylem vessel, differentiating xylem and phloem, according to public data [1].

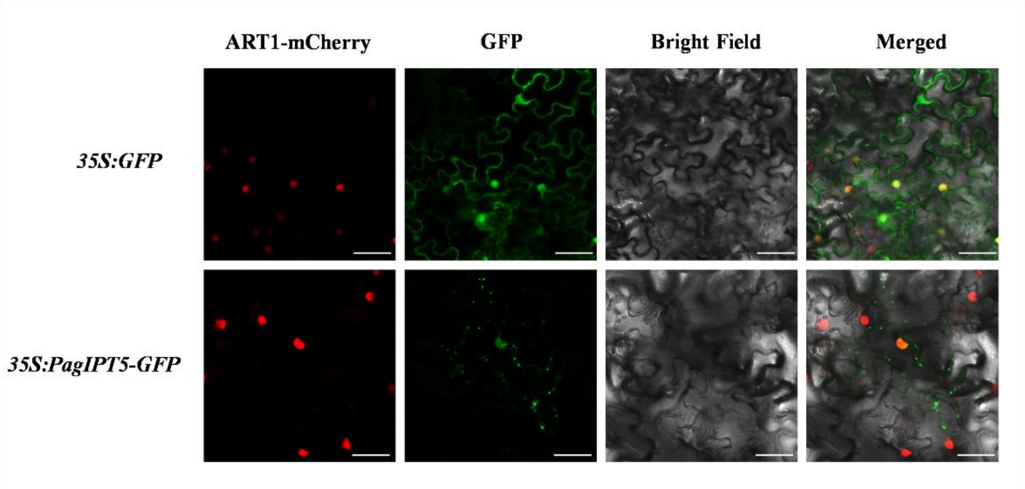

Supplementary Figure S2. Subcellular localization of the protein encoded by *PagIPT5*
Expression of the PagIPT5-GFP fusion protein in tobacco mesophyll cells. Scale bar = 50 μm

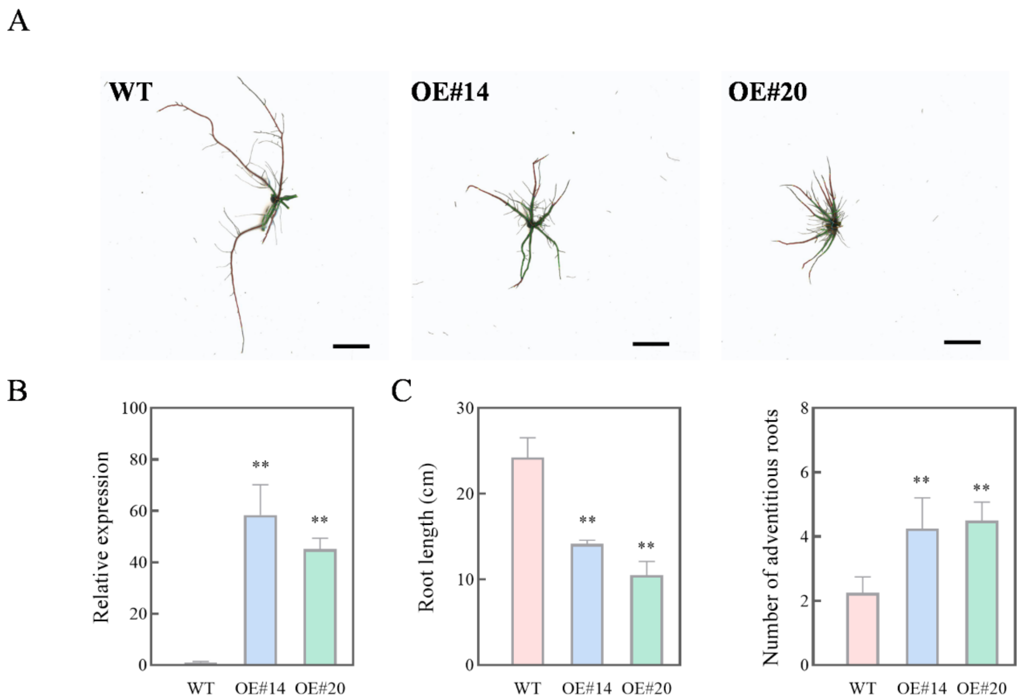

Supplementary Figure S3. Root growth parameters in *PagIPT5* overexpression plants

(A) Root scanning images of *PagIPT5* overexpression and wild-type tissue-cultured plants. Scale bar = 0.5 cm. (B) Detection of *PagIPT5* transcriptional levels in transgenic lines ($n \geq 3$). (C) Quantification of root length ($n = 4$) and adventitious root number ($n = 4$). Data are represented as mean ± SD. $**P \leq 0.01$; Student's *t*-test.

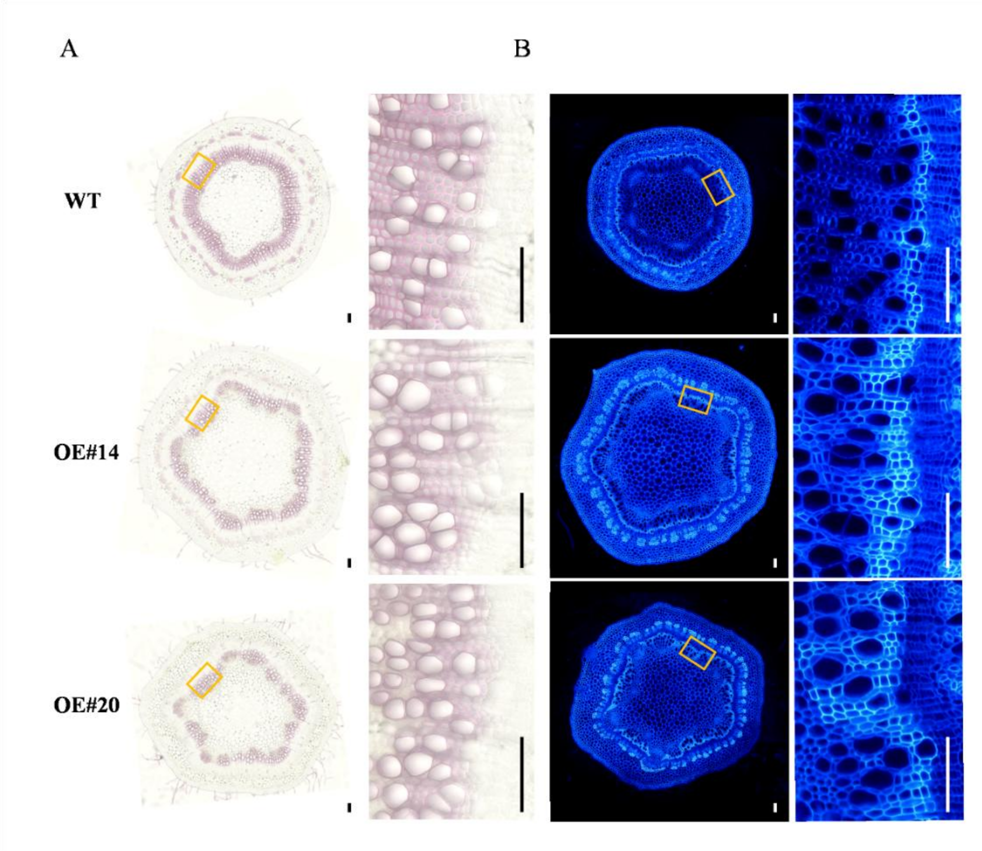

Supplementary Figure S4. Qualitative detection of lignin and cellulose in *PagIPT5* overexpression plants

(A–B) Phloroglucinol and CFW histochemical staining of vascular xylem in the 10th internode of *PagIPT5* overexpression and wild-type soil-grown plants. Scale bar = 50 μm

References

1. Shi, R.; Wang, J.P.; Lin, Y.-C.; Li, Q.; Sun, Y.-H.; Chen, H.; Sederoff, R.R.; Chiang, V.L. Tissue and cell-type co-expression networks of transcription factors and wood component genes in Populus trichocarpa. *Planta* **2017**, *245*, 927-938
